# Identifying High-Risk Events for COVID-19 Transmission: Estimating the Risk of Clustering Using Nationwide Data

**DOI:** 10.3390/v15020456

**Published:** 2023-02-06

**Authors:** Minami Ueda, Katsuma Hayashi, Hiroshi Nishiura

**Affiliations:** School of Public Health, Graduate School of Medicine, Kyoto University, Yoshida-Konoe-cho, Sakyo-ku, Kyoto 606-8501, Japan

**Keywords:** severe acute respiratory syndrome coronavirus 2 (SARS-CoV-2), epidemiology, observational study, disease outbreak, contact tracing, risk assessment

## Abstract

The transmission of severe acute respiratory syndrome coronavirus 2 (SARS-CoV-2) is known to be overdispersed, meaning that only a fraction of infected cases contributes to super-spreading. While cluster interventions are an effective measure for controlling pandemics due to the viruses’ overdispersed nature, a quantitative assessment of the risk of clustering has yet to be sufficiently presented. Using systematically collected cluster surveillance data for coronavirus disease 2019 (COVID-19) from June 2020 to June 2021 in Japan, we estimated the activity-dependent risk of clustering in 23 establishment types. The analysis indicated that elderly care facilities, welfare facilities for people with disabilities, and hospitals had the highest risk of clustering, with 4.65 (95% confidence interval [CI]: 4.43–4.87), 2.99 (2.59–3.46), and 2.00 (1.88–2.12) cluster reports per million event users, respectively. Risks in educational settings were higher overall among older age groups, potentially being affected by activities with close and uncontrollable contact during extracurricular hours. In dining settings, drinking and singing increased the risk by 10- to 70-fold compared with regular eating settings. The comprehensive analysis of the COVID-19 cluster records provides an additional scientific basis for the design of customized interventions.

## 1. Introduction

As of March 2022, 470 million cases of coronavirus disease 2019 (COVID-19) and 6 million deaths had been recorded worldwide since the start of the pandemic [1]. During the early phases of this pandemic, many countries enforced radical countermeasures, such as lockdowns, to reduce the spread of COVID-19. Although such drastic interventions to reduce contacts have been proven effective in substantially reducing the reproduction number [2], they can have devastating effects on national economies [3] and people’s overall health outcomes, including social well-being [4]. Due to the widespread vaccine-induced and naturally acquired immunity in populations worldwide, more countries are seeking a balance between controlling COVID-19 and boosting socioeconomic activities.

The COVID-19 epidemic levels in Japan have been maintained at relatively lower levels than those in Western countries, with a less than 2% cumulative risk of confirmed infection by the end of 2021. Japan is one of the few countries that has accumulated data on clusters since the early phases of the pandemic [5] by focusing on the prevention of clusters via retrospective contact tracing. Since the transmission of severe acute respiratory syndrome coronavirus 2 (SARS-CoV-2) is highly dispersed [6,7,8,9,10,11,12], meaning that a small fraction of infected people produce secondary cases, the chain of transmission can effectively be contained by identifying these “super-spreading” individuals. Consequently, retrospective contact tracing, also known as the “backward tracing” approach, is an effective outbreak control measure that can be used to ascertain clusters involving asymptomatic infections [13]. The expert committee on COVID-19 prevention in Japan analyzed the cluster surveillance data in early 2020 and proposed the concept of the “3Cs” (i.e., closed spaces, crowded places, and close contact settings), which are conditions where infections are likely to occur [14]. Public health and social measures in Japan initially requested that social contacts and movements be uniformly reduced in the form of a state of emergency (SoE). As an alternative measure to the SoE, pre-emergency measures (PEMs) were introduced in early 2021, in which interventions were more customized and focused on high-risk settings [15] that are likely to meet the 3Cs concept. This included settings such as eating and drinking establishments that serve alcohol. PEMs allowed the local governor of the target area to decide which sub-regional areas would be subject to these customized interventions [15].

In support of the 3Cs concept, published studies have demonstrated that SARS-CoV-2, which causes COVID-19, is primarily transmitted through exposure to respiratory fluids via inhalation, deposition, and contact with contaminated surfaces or objects [16,17,18]. Detailed environmental factors in COVID-19 transmission include enclosed spaces with inadequate ventilation and poor air quality [14,16,19,20,21,22,23], increased exhalation of respiratory fluids [16,24,25,26,27,28,29,30,31], crowded and close contact settings [14,19,23,32], and prolonged exposure [16,19,33]. Studies have also identified specific occasions and workplaces where infections are likely to occur: health care and long-term care facilities [34,35,36,37] and the hospitality and entertainment sectors (i.e., bars where eating and drinking take place) [22,32,35,38,39,40,41].

On the basis of evidence and expert opinions, the Infectious Diseases Society of America has qualitatively classified daily activities into high-, medium-, and low-risk categories [42]. However, such an assessment remains narrative. Studies on quantitative risk assessment have mostly been restricted to a specific activity category, such as eating and drinking [39], attending schools [43], and workplaces [44]. A test-negative study confirmed an elevated risk of infection in eating and drinking establishments [32], but the comparison was made between positives and negatives among those who attended healthcare establishments, and the sample size was limited. A modeling study showed a notably high infection risk in restaurants during the process of reopening [40], but the risk estimates relied on model fitting procedures using aggregated anonymized location data. A study analyzing surveillance and clinical data showed that entertainment settings, especially bars, have the longest cascade of cases [41]. However, this analysis was based on descriptive statistics and did not analyze the risk of clustering per event, which takes into account the number of potential chances of infection. To the best of our knowledge, no study has comprehensively quantified and compared the risk of clustering that accounts for the patterns of use (i.e., duration, frequency, and the number of users) in diverse daily activities using cluster records in which linked cases were identified through forward and backward tracing.

This study aimed to estimate the risk of COVID-19 clustering in a wide range of daily activities and to identify high-risk conditions of clustering by comparing the risk across different types of settings, including schools and eating and drinking establishments.

## 2. Materials and Methods

### 2.1. Cluster Surveillance Data

In this study, we used the incidence of COVID-19 clusters in Japan, which relies on the systematic collection of publicly available information on cluster events. A team commissioned by the Ministry of Health, Labour, and Welfare (MHLW) routinely collected published information on clusters from local health authorities and news articles in the media. This team consisted of full-time researchers from the National Institute of Infectious Diseases (NIID), Tohoku University, and Hokkaido University. The records were routinely updated to reflect the latest information on the identified clusters. We analyzed the entire period of cluster surveillance from June 2020 to June 2021. The SoE, the Japanese implementation of public health and social measures, was declared for some prefectures (a maximum of 11/47 prefectures) in the latter part of the study period. The number of reported clusters and cases by month starting in June 2020, along with the range of SoEs, is shown in Figure S1. Only one variant of concern became predominant during the study period, and it occurred in the wave from March to June 2021 caused by the Alpha variant (B.1.1.7). When we compared the trend of the number of reported cases between the Alpha variant wave and the former wave (October 2020 to February 2021), we did not observe any significant difference in the trend of the number of reported clusters. We believe that the effect of the variant’s predominance on the risk of clustering is negligible because the transmission of concerned variants has remained overdispersed. Additionally, a proportion of the population was vaccinated in the latter part of the study period. The vaccination program in Japan began on 17 February 2021 and prioritized healthcare workers, followed by individuals aged 65 years and older from 12 April. The national vaccination uptake rate was only 10.62% at the end of June 2021 (Figure S2). Therefore, we believe that the effects of herd immunity on transmission patterns are negligible.

We defined a *cluster* as “a group of infected individuals who were identified through one or more links of infection and tested positive”, with usually five or more cases that emerged from the same environmental setting [45,46]. Following a guideline presented by the MHLW and the NIID, clusters were identified through contact tracing—combining both forward and backward tracing—by local health authorities with the legally mandated cooperation of infected individuals. A total of 468 main offices of local health authorities are located throughout Japan in every prefecture. Those who were acknowledged as close contacts from contact tracing were additionally tested. Positive cases were confirmed via a reverse transcription-polymerase chain reaction (RT-PCR) test or an antigen test (including both a qualitative test based on immunochromatography and a quantitative test based on the chemiluminescence enzyme immunoassay [CLEIA] method). Reporting of positive cases to the MHLW was mandated by the infectious disease control law in Japan [47]. Even when an antigen test resulted negative, an RT-PCR test was additionally conducted at a physician’s discretion for confirmation if the patient was suspected of being infected. Although COVID-19 screening (which includes testing for people without symptoms) in elderly care facilities and health institutions was approved by the government health authority in 2021, it has not been conducted in a systematic and regular manner. However, the members of facilities were prioritized for testing if a case was reported in elderly care facilities. Based on publicly available data from the MHLW, the majority of the tests conducted were performed using the RT-PCR method (Figure S3).

The original dataset included the following information for each record of a cluster: type of setting (primary and secondary), prefecture, name of the cluster, number of cases in the cluster, occurrence date, and notes. Information on the size and area of the location was not available in the dataset.

Prior to the analysis, we additionally annotated the type of setting in each cluster record by reclassifying these into primary, secondary, tertiary, and quaternary categories. This classification was based on the following information: the original classification, the unique name of the cluster, and additional information found in the notes (Figure S4). We used information obtained from search engines, online maps, and news articles to judge the type of setting when the information provided in the original record was inadequate. After the annotation process was completed, we finalized the dataset so that one record corresponded to one specific type of setting. We selected records with the following types of settings for analysis: elderly care facility, welfare facility for people with disabilities, hospital, clinic, dental clinic, serviced entertaining bar and escort club, alcohol-serving eating and drinking establishment, eating and drinking establishment with karaoke, restaurant, university, high school, middle school, elementary school, child welfare facility, kindergarten, cram school, workplace, police station, local government office, sports facility, fire station, theater, and beauty salon or barber (Figure S5). “Serviced entertaining bars and escort clubs” included facilities such as pubs, hostess bars, and snack bars, where staff primarily entertained customers while drinking in close proximity. These facilities are registered as “types 1, 2, and 3” under the Entertainment Business Act. An “alcohol-serving eating and drinking establishment” included facilities categorized as a “late-night liquor service restaurant”, which comprise casual bars such as “Izakaya” (Japanese-style bars) where customers enjoy eating and drinking by themselves or with friends. A “restaurant” encompassed the remaining restaurants and coffee shops used for usual dining occasions other than the preceding two types of setting. With regard to educational settings, “child welfare facilities” included nursery schools, where children aged 0 to 6 years are looked after during the day while their parents work. A kindergarten is a facility similar to a nursery school but with a shorter duration of use and limited age groups from 3 to 6 years old. We calculated the sum of cluster reports for each setting type, represented as $c_{x}$, where x represents the type of setting (x=1,2,⋯,23) (Table S1).

### 2.2. Event Frequency and Users

Additional data on events and types of settings were used to calculate the risk of clustering per event (Table 1). For each type of setting, we defined a concept named *event*, which is a unit of use of the facility each day. An example of this concept is that the definition of an event for a high school was “attending school” for a duration of 8 h per day, which occurs every weekday except during vacations.

We calculated the “total number of events” during the observation period by multiplying the number of days of event $d_{x}$ by the number of facilities $f_{x}$, representing how many days the facilities were open or used during the corresponding period. The number of facilities $f_{x}$ was obtained or calculated using vital statistics and survey-based statistical information. We used the latest statistics available at the point of analysis. The number of event days $d_{x}$ was calculated by summing the days for every month according to business and academic calendars.

Two indices—the average number of users per event ${\bar{u}}_{x}$ and the duration of the event $t_{x}$—were used as the denominator to standardize the activity-dependent risk of clustering between different setting types. We lacked information on the changes in the number of users. Therefore, we used the average value from the pre-COVID period, which was obtained or calculated from statistics, for most of the setting types (other than sports facilities, serviced entertainment bars and escort clubs, alcohol-serving eating and drinking establishments, and restaurants). We have made the assumption that the number of users in other types of settings (i.e., schools, workplaces, and welfare facilities) did not significantly change owing to the nature of establishments. The SoE implemented during the study period was not as powerful as the first SoE in April 2020, which included uniform school closure in the nation. We set assumptions on the duration of the event, assuming a typical length of use if relevant statistics were unavailable. Details of the calculation for each parameter are available as part of the Supplementary Material (Text S1).

### 2.3. Estimating the Risk of Clustering

We calculated three types of the “activity-dependent cluster infection risk index” by dividing the sum of cluster reports by three types of denominators. Given that the patterns of use are different for every type of setting (e.g., people stay longer at schools and workplaces than at restaurants), the three indices have different values in the denominator to allow for the comparison of risks from multiple aspects. $\gamma_{x}$ (reports/event)—representing the risk of clustering per event per facility, without adjustment for the number of users per event and duration per use—was calculated by dividing the total number of cluster reports by the total number of events. ${\gamma_{x}}^{\prime }$ (reports/[person·event])—representing the risk per event user—was calculated by adjusting $\gamma_{x}$ for the number of users per event. This index identifies the risk of clustering if the same number of people engage in respective events in each establishment, which is likely to be the most common interest for the general public as it represents the risks they are exposed to for an ordinary usage occasion. ${\gamma_{x}}^{\prime \prime }$ (reports/[person·event·hour])—representing the risk of clustering per event user hours—was calculated by adjusting $\gamma_{x}$ for the number of users per event and duration per use. This index identifies the risk of clustering if the number of users and the duration of usage were equivalent across all establishment types.

We calculated these risk estimates as:

$$\begin{array}{cc} \gamma_{x} & =\frac{c_{x}}{\Sigma_{j=1}^{f_{x}}\left(\Sigma_{i=m}^{n}d_{x_{i}}\right)}\\ {\gamma_{x}}^{\prime } & =\frac{c_{x}}{\Sigma_{j=1}^{f_{x}}\left(\Sigma_{i=m}^{n}d_{x_{i}}\right)\cdot {\bar{u}}_{x}}\\ {\gamma_{x}}^{\prime \prime } & =\frac{c_{x}}{\Sigma_{j=1}^{f_{x}}\left(\Sigma_{i=m}^{n}d_{x_{i}}\right)\cdot {\bar{u}}_{x}\cdot t_{x}} \end{array}$$

In the equations, i indicates the month, where the lower limit m and upper limit n correspond to June 2020 and June 2021, respectively. The numerator and denominator for each calculation are shown in Table S2. We calculated the 95% confidence interval (CI) using the Agresti–Coull method [48].

We also calculated the rate ratio (RR) of ${\gamma_{x}}^{\prime }$, the activity-dependent risk of clustering (adjusted for the number of users) among two categories consisting of similar types of setting—educational and dining facilities—to understand the relative chance of clustering. ${\gamma_{x}}^{\prime }$ was used for the evaluation of RR for two reasons: (a) it represents the risks that users are exposed to for an ordinary usage occasion; (b) the study aimed to provide the best robust estimate possible without introducing an additional assumption for the duration of usage, as we did in ${\gamma_{x}}^{\prime \prime }$. In educational settings, the types of settings included an elementary school, middle school, high school, university, child welfare facility, kindergarten, and cram school. Elementary school was set as the baseline for calculating the RR. With regard to dining, the types of settings included serviced entertaining bars and escort clubs, eating and drinking establishments with karaoke, alcohol-serving eating and drinking establishments, and restaurants. The restaurant was set as the baseline. The CI of the RR was derived using the Wald method. We also calculated the risk difference using the same baseline as a supplemental analysis. All calculations were performed using R software version 3.6.3 [49].

### 2.4. Sensitivity Analysis on the Estimates

We conducted a sensitivity analysis of the estimates by varying the parameters within the possible range. The range of each parameter is shown in Table S3. Using the Latin hypercube sampling method, we randomly generated 10,000 pairs of parameters, assuming that they followed a uniform distribution in the defined range. We then calculated the risk index (${\gamma_{x}}^{\prime }$), the activity-dependent risk of clustering adjusted for the number of users, for each type of setting using each pair. This process resulted in the distribution of the risk index for each type of setting. Additionally, by using the denominators of the types of settings that were derived from 10,000 parameter pairs, we calculated the RR for each type of setting in educational and dining facilities. The process similarly resulted in the distribution of RR for each type of setting.

### 2.5. Simulation-Based Bias Assessment

We performed a simulation-based assessment to evaluate the potential reporting bias in the cluster surveillance data. Specifically, we evaluated our estimate on the rate ratio of the risk of clustering by comparing it with an estimate based on the values from an external study that is not likely to be influenced by reporting biases. We used values from a reference study that assessed the risks of reopening using a fine-grained dynamic mobility network using geolocation data constructed from mobile phones in the USA [40]. They estimated the additional infections for each setting (point of interest; POI) when a particular POI was reopened while other POIs continued to experience a limited level of activities. Among the POIs in the reference study, we focused on “full-service restaurants” and “limited-service restaurants” since we could consider that they respectively correspond to “alcohol serving eating and drinking establishment” and “restaurant” in our study, according to the definitions of the North American Industry Classification System.

Using our cluster surveillance data, we first calculated a rate ratio, separate from the one described earlier, that can be compared with the rate ratio estimated using the values from the reference study. For such convenience, we calculated a provisional index by dividing the number of clusters by the number of facilities. In other words, the index indicated the number of clusters normalized by the number of facilities, which is equivalent to POI in the reference study. By dividing this index for “alcohol serving eating and drinking establishment” by that for “restaurant”, we derived a rate ratio that is subject to the bias assessment. We computed the 95% CI using the Agresti–Coull method.

We then calculated the rate ratio of the risk of clustering using the values extracted from the reference study. From Extended Data Figure S5c of the reference study [40], we manually extracted the estimates of additional infections per 100 k patients compared with not reopening, normalized by the number of POIs, using WebPlotDigitizer [50] and fitted the estimates to normal distributions from the median, 25th percentile, and 75th percentile. For each setting, we obtained the distribution of the number of clusters through Monte Carlo simulation by dividing the fitted distribution of the number of additional infections by the distribution of the size of clusters observed in our cluster surveillance data. The mean size of clusters observed in our cluster surveillance dataset was 8.91 and 6.67 for “alcohol serving eating and drinking establishment” and “restaurant”, respectively. Just as the provisional index that we calculated from our data indicates, this index indicates the number of clusters normalized by the number of POIs. We used 20,000 samples, respectively, in the process of numerical computation. From the simulated distributions of the number of clusters, we obtained the distribution of rate ratio by dividing the value for “full-service restaurant” by “limited-service restaurant”. We conducted parametric bootstrapping from 20,000 samples and calculated the mean and its 95% CI using the bootstrap percentile method.

### 2.6. Evaluation on the Consistency of Reporting througout the Study Period

Additionally, we conducted a separate analysis to evaluate the consistency of reporting throughout the study period. We first calculated the proportion of the number of clusters for each type of setting until and after the second wave and determined the difference in proportions between the periods. Here, we defined the end of the second wave as the end of September 2020.

## 3. Results

**Figure 1:**
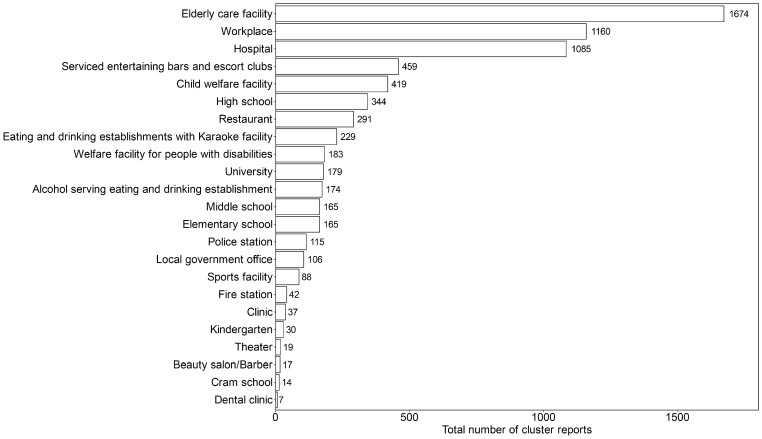
Total number of cluster reports by the type of setting from June 2020 to June 2021. The types of settings were annotated for each record of clusters. The figure shows the total number of cluster reports from June 2020 to June 2021.

A total of 7002 clusters were reported in 23 types of settings from June 2020 to June 2021. The average size of clusters included in the dataset was 13.31 people per cluster. Overall, the incidence of cluster reports was proportional to the incidence of confirmed cases in Japan (correlation coefficient of 0.92, Figure S1). Generally, clusters predominantly occurred in elderly care facilities, workplaces, and hospitals, with 1674, 1160, and 1085 reports, respectively (Figure 1). Among all types of settings, elderly care facilities had the largest number of clusters, with 1674 total reports and a monthly peak of 370 reports in January 2021 (Table S1).

Among university clusters, in-depth exploration, in which we examined the settings further in detail based on the name of the cluster and notes, showed that 46.4% were sports-related (i.e., athletics clubs and other extracurricular activities). Only 0.56% of school clusters occurred within the classroom or during classes. Among high school clusters, 8.1% were identifiable as sports-related. Apart from sports-related clusters, another 8.7% of clusters in high schools were related to extracurricular activities; however, details regarding the types of activities were unknown.

### 3.1. Activity-Dependent Cluster Infection Risk Index

**Figure 2:**
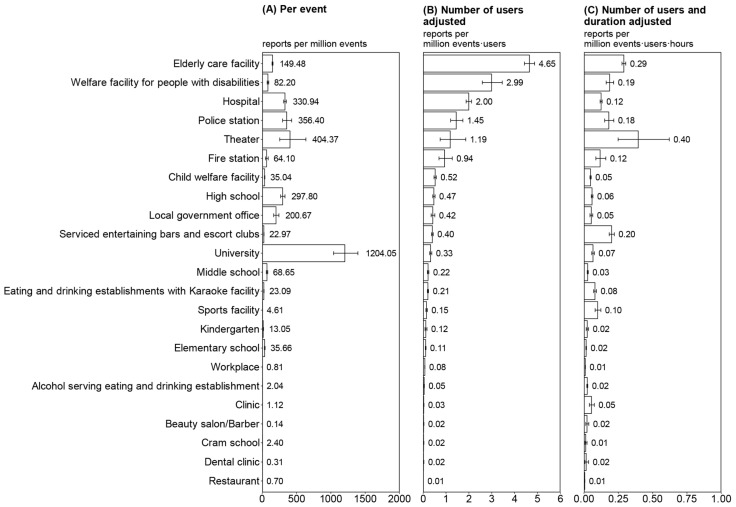
Activity-dependent risk of clustering from June 2020 to June 2021. In each type of setting, we defined event, which represented a typical daily use unit, (i.e., going to school, working at the office, and eating at a restaurant). (**A**) Plot of $\gamma_{x}$, which represents the risk per event without adjustment for the number of users and duration per event. (**B**) Plot of ${\gamma_{x}}^{\prime }$, which represents the risk when controlling for the number of users. (**C**) Plot of ${\gamma_{x}}^{\prime \prime }$, which represents the risk, controlling for the number of users and duration of use. The 95% confidence interval was calculated using the Agresti–Coull method.

With regard to the activity-dependent risk of clustering without any adjustment ($\gamma_{x}$), university settings showed the highest risk, with 1204 reports per million events (95% CI: 1039–1394) (Figure 2A). This was followed by theaters (404; 95% CI: 254–636), police stations (356; 95% CI: 297–428), hospitals (331; 95% CI: 312–351), high schools (298; 95% CI: 268–331), and local government offices (201; 95% CI: 166–243).

When adjusted for the number of users per event (${\gamma_{x}}^{\prime }$), the risk of clustering at elderly care facilities, welfare facilities for people with disabilities, and hospitals were prominent, with 4.65 (95% CI: 4.43–4.87), 2.99 (95% CI: 2.59–3.46), and 2.00 (95% CI: 1.88–2.12) reports per million event-users, respectively (Figure 2B). This was followed by police stations, theaters, and fire stations. Restaurants had the lowest risk, with 0.01 reports per million event users.

When activity-dependent risk was adjusted for the number of users and duration of the event (${\gamma_{x}}^{\prime \prime }$), elderly care facilities, welfare facilities for people with disabilities, and serviced entertainment bars and escort clubs showed comparably high levels of risk (Figure 2C). Theaters had the highest risk with 0.40 reports per million event-user-hours, and the 95% CI ranged broadly from 0.25 to 0.62. When the same number of individuals was assumed to have used each facility for the same duration, these facilities were regarded as having a high risk.

### 3.2. Rate Ratios of Activity-Dependent Risk

**Figure 3:**
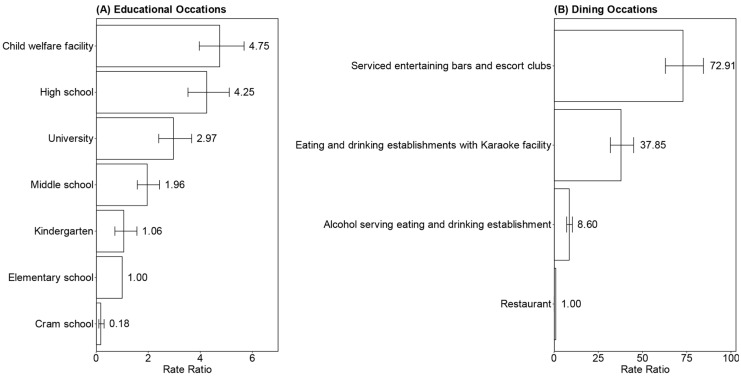
Rate ratio of the activity-dependent risk of clustering (adjusted for the number of users) in educational and dining settings. The rate ratio of the activity-dependent risk of clustering (adjusted for the number of users) ${\gamma_{x}}^{\prime }$ was calculated among two categories consisting of similar types of settings: (**A**) educational and (**B**) dining settings. Elementary school and restaurants were considered the baseline, respectively. The 95% confidence interval was calculated using the Wald method.

Among educational facilities, the RR of clustering was frequently higher in older age groups, excluding child welfare facilities (Figure 3). Child welfare facilities had the highest RR of 4.75 (95% CI: 3.96–5.68), followed by high school (4.25; 95% CI: 3.53–5.11), university (2.97; 95% CI: 2.40–3.67), middle school (1.96; 95% CI: 1.58–2.43), and kindergarten (1.06; 95% CI: 0.72–1.57). Cram school was the only type of setting with a lower risk than elementary school.

With regard to dining, serviced entertainment bars and escort clubs had a high risk of clustering with a RR of 72.9 (95% CI: 62.95–84.44), followed by eating and drinking establishments with karaoke (37.85; 95% CI: 31.83–45.00) and alcohol-serving eating and drinking establishments (8.60; 95% CI: 7.12–10.37) compared with ordinary restaurants (baseline). The risk differences for types of settings in both categories is shown in Tables S4 and S5.

### 3.3. Sensitivity Analysis of the Risk Index and RR

The results of the sensitivity analysis of the risk index and RR are shown in Figures S6–S8. Each of the figures shows the distribution of each index when the parameters were altered within the predefined possible range. The results of the sensitivity analysis were generally consistent with the original estimates shown in Figure 2 and Figure 3. The mean of the randomly generated 10,000 samples is shown in the top right corner of each panel. The original estimates were within a reasonable range of the estimated distribution.

### 3.4. Simulation-Based Bias Assessment

The provisional index for our cluster surveillance data, used in the process of bias assessment, was 658.20 and 227.38 for “alcohol serving eating and drinking establishment” and “restaurant”, respectively. This resulted in the rate ratio, subject to the bias assessment, for “alcohol serving eating and drinking establishment” compared with “restaurant” to be 2.89 (95% CI: 2.40–3.49).

**Figure 4:**
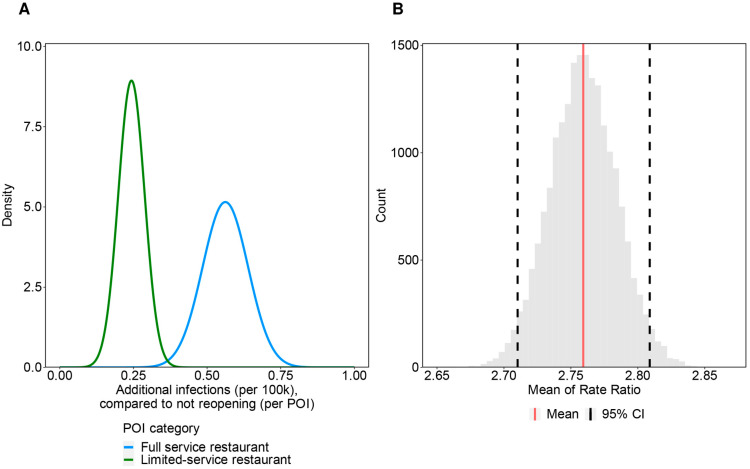
Result for simulation-based bias assessment: (**A**) Fitted distribution of additional infections compared to not reopening per POI; (**B**) Bootstrapped mean of the rate ratio between “full service restaurant” and “limited-service restaurant” in the reference study. (**A**) shows the fitted distribution of additional infections (per 100 k) compared to not reopening, normalized by the number of POI. The distributions were fitted from median, 25th percentile, and 75th percentile in the original figure of the reference study assuming that they follow normal distribution. (**B**) is the distribution of the bootstrapped mean of rate ratio. Red line shows the mean (2.76) and dotted line shows the 95% CI (2.71–2.81) computed by bootstrap percentile method.

The fitted distributions of the number of additional infections (per 100 k) compared with not reopening, normalized by the number of POIs, for “full service restaurant” and “limited-service restaurant” are shown in Figure 4A. Using the simulated distribution of the number of clusters in the reference study data, we estimated the mean of the rate ratio for “full service restaurant” compared with “limited-service restaurant” to be 2.76 (95% CI: 2.71–2.81). Figure 4B shows the distribution for the bootstrapped mean of the rate ratio. The estimate from the reference study data provided a consistent value for our estimate, with overlaps in the 95% CI.

### 3.5. Evaluation on the Consistency of Reporting throughout the Study Period

In terms of the difference in the proportion of the number of clusters for each type of setting until and after the second wave, schools and care facilities experienced a particular increase in their proportions: elderly care facilities (+11.71 percentage point [pp]), high schools (+2.24 pp), elementary schools (+1.12 pp), and welfare facilities for people with disabilities (+1.07 pp). In contrast, eating and drinking facilities and other settings in the general community observed a particular decrease in their proportions: serviced entertainment bars and escort clubs (−4.58 pp), restaurants (−3.54 pp), alcohol-serving eating and drinking establishments (−2.92 pp), workplaces (−1.86 pp), and eating and drinking establishments with karaoke (−1.19 pp). The values indicate the transition of the major settings where clustering has occurred: settings with positive values experienced a surge of clusters in the later half of the epidemic. The trends generally matched with preceding reports suggesting that clusters initially occur in high-risk settings such as alcohol-serving eating and drinking establishments, propagate to the general community, including schools, and eventually reach the elderly care facilities that are located at the end of the local transmission chain [24,37]. Consistent reporting of clusters is also supported by the comparison of the number of reported clusters and cases by month, as shown in Figure S1.

## 4. Discussion

Using unique cluster surveillance data from Japan, we investigated the activity-dependent risk of COVID-19 clustering by analyzing the cumulative frequency of cluster reports. Our risk calculation indicated that care facilities posed the highest risk of clustering per event-user among all examined facilities. A substantial risk with drinking and singing (karaoke) was also observed in dining settings, with an RR 10- to 70-fold greater than usual eating occasions at restaurants. In educational settings, the risk of clustering in child welfare facilities and high schools was nearly four times that of elementary schools.

Our findings could help provide a better understanding of the underlying factors that lead to the clustering of COVID-19 cases. The different levels of clustering among diverse types of settings—primarily caused by differences in characteristics, environments, duration, and frequency of the activities held in each setting—further support the scientific notion of “focused interventions”. These interventions have partly been implemented in Japan as PEMs. In particular, the focus on countermeasures on eating and drinking establishments in PEMs is quantitatively supported by our results. With presentations of the risks as numerical estimates, interventions, and resource allocations can be further optimized.

The increased risk in care facilities (i.e., elderly care facilities, welfare facilities for people with disabilities, and hospitals), which is in line with existing studies [34,35,36,37], could reflect differences in the duration and quality of contact. As we have assumed in Table 1, many users *live* in these facilities, meaning that there would be prolonged contact. Moreover, owing to the need for physical services provided by caregivers, close contact occurs more often than in other settings. Our findings showing that care facilities are vulnerable to clusters imply that prevention measures for these establishment types should be strengthened. Furthermore, our findings also support the prioritization of vaccination among older people and healthcare professionals, in line with previous evidence [51].

With regard to educational facilities, an overall elevation in the risk of clusters among older age groups is consistent with prior evidence indicating that the spread of SARS-CoV-2 can occur more easily in high schools than in primary schools [52]. The risk of infection could have increased owing to the tendency that schools with older students have larger class sizes, and the frequency and range of social contacts are higher in older age groups [53]. Individuals in older age groups commonly take classes at cram schools with students from other schools, commute to school by train, and participate in extracurricular and volunteer activities in the community outside of school. In-depth analysis of school clusters suggests that the principal causes of infection involve sports- and extracurricular-related activities. As prior research has identified a low risk of SARS-CoV-2 transmission through interactions in close proximity during contact sports [54], settings outside of actual sports practice, such as locker rooms, dormitories, or eating and drinking after the activity, may be the primary source of clustering. While further research is required to pinpoint the exact cause of COVID-19 infections in educational facilities, classes themselves may not necessarily present a high risk of clustering. The difference in risks between child welfare facilities and kindergarten, where the age range of users is similar, could be due to several factors. First, the duration of use is generally longer in child welfare facilities, with children remaining at the facility until their parents come to pick them up after work. In contrast, children in kindergarten usually go home much earlier. Furthermore, the parents’ working status could be related to the increased risk in child welfare facilities. For the reason that parents who leave their children in child welfare facilities work outside the home, in contrast to the parents of children in kindergarten, the chance of the former parents becoming infected could be higher. This situation could ultimately lead to the virus spreading at home and to facilities where the child spends time. While the extent is unknown, the wider range of ages in child welfare facilities (0 to 5 years old compared with 3 to 5 years old for kindergarten) could also contribute to the increased infection risk.

Our findings provide evidence that serviced entertainment bars and escort clubs and eating and drinking establishments with karaoke have a significantly greater risk of clustering than regular eating and drinking settings. The content of activities determines the quality of contact and could thus characterize the risks as follows. First, intoxication can induce behaviors that are likely to result in a higher rate of infection. As reported as a concern among staff working at serviced entertainment bars and escort clubs, individuals are likely to be less conscientious about infection prevention measures in an intoxicated state [55]. Proper wearing of masks can easily be forgotten, and individuals are likely to speak in a louder voice, which causes more emission of respiratory droplets than usual [56,57]. Additionally, singing (karaoke) can further intensify the risks. Although our results should be interpreted with caution because of the population-level study method, they suggested that singing was five times more likely to cause clustering than situations involving alcohol consumption. There are numerous reports of clusters arising from group singing events, and singing is known to cause more dispersion of aerosols [56]. Notably, clusters in eating and drinking establishments with karaoke have most frequently occurred on a specific occasion called “*Hiru-kara*” in Japanese. Unlike usual karaoke settings in karaoke boxes, *Hiru-kara* is a unique form of karaoke in which older adults remain for an extended period during the daytime and into the evening to enjoy singing, eating, and talking with each other. On these occasions, ventilation, which is considered an environmental factor in infection [14,16,19,22,23], could have been insufficient compared with the usual karaoke boxes, which have their own guidelines for infection prevention that were decided under the supervision of public health experts. Poor ventilation is also often observed in serviced entertainment bars and escort clubs. The design structure of these venues is stipulated by law so that the interior cannot be easily seen from the outside, which prevents a sufficient flow of air even when ventilation is attempted [55]. Although our RR results were significantly high and could be interpreted as an overestimation, there have been numerous COVID-19 cases from settings consisting of the above-mentioned factors (i.e., singing and drinking) [24], which support the validity of our estimates. The results of the simulation-based bias assessment, in which we compared the risk ratio using values from a mobility-data-based study that was unlikely to have been influenced by reporting bias, indicated that our estimates possess a comparable level of limited reporting bias within the best extent that we could assess. The result of the difference in the proportions of the clusters until and after the second wave indicates that the reporting was conducted consistently, even amid changes in major settings where clusters frequently occur. The risk for “serviced entertaining bars and escort clubs” could potentially be higher than our estimate because people are likely to be more hesitant to disclose that they have been to such establishments.

We must acknowledge four major limitations of this study. First, while we have shown that the data has unlikely been influenced by the reporting bias as far as we could assess, we still cannot deny the possibility for further reporting and ascertainment biases in the results, owing to the design of data collection. The study investigated clusters that were reported to the Japanese health authorities and were subsequently reported publicly (e.g., via the media). The results regarding types of settings, such as schools and public offices (police, fire stations, and local governments), are more likely to reflect the actual risk than other types of settings because more clusters in these settings are likely to be reported and covered in the media. The results regarding care facilities can be regarded similarly because the testing frequency is commonly more intensive than that in other types of settings. Children are likely to be asymptomatic or mildly symptomatic even when infected with COVID-19. Therefore, the number of clusters in schools and child welfare facilities could have been underreported, resulting in the underestimation of risks. Additionally, the transmission dynamics have altered over time owing to multiple factors after the study period (i.e., emergence of variants, progress of immunization, changes in policies). Especially in the early stages of 2022, child welfare facilities and schools have become one of the major settings of clustering (Figure S2), because progress in immunization has decreased the risk in health care and elderly facilities [51]. Furthermore, the limited testing capacity in Japan [58] may have affected our estimates on the risk of clustering. Taking into consideration the results of a serological survey in Japan [59], we believe that the risk of clustering could be four times that of the original estimate. Even with such potential biases, the present study provided a comprehensive overview of differences in the risk of clustering for most activities in which risk is a concern. While the absolute value of the estimates may need further confirmation from additional studies, we consider that the relative levels of risks in this study can contribute to real-world decision making during the pandemic to effectively allocate limited resources. It has to be noted that risks for each setting may change due to attributable factors such as policy restrictions, interventions, vaccination rates, participant risk behaviors, and compliance with the preventive measures. Second, limitations in the denominator of risks should be discussed, especially for the use of average values from census data and the time dependence of these values. Due to the SoE’s declaration to reduce contact on a voluntary basis, the actual activity patterns might have varied substantially in a dynamic manner over time. We also note that the assumptions for constant school attendance throughout the period lacked quantitative support. Third, most clusters in theaters did not involve audiences but were mainly workplace clusters. Most infected people within theater clusters were performers and staff, and only a few reports have indicated infections among audience members. Fourth, owing to our definition of clusters, local settings with frequent transmission but involving a small number of people (e.g., family events or activities among a small group of friends) were not captured in the dataset. Therefore, our dataset could not identify household transmission risks. The findings from our study should be interpreted with caution because of the inherent limitations listed above.

## 5. Conclusions

Through a systematic assessment of the activity-dependent risk of COVID-19 case clustering, this study shows that care facilities pose the highest risk of clustering per event user among all facilities. Additionally, drinking and singing in settings accompanied by eating significantly increases the risk of infection by 10- to 70-fold compared with eating in an ordinary restaurant. Among educational facilities, the infection risk is proportionately greater in older age groups and frequently associated with sports and extracurricular activities. The present study is the first to comprehensively analyze COVID-19 cluster records accumulated in Japan, in which clusters were identified via backward and forward contact tracing. By conducting similar surveys during the rest of the pandemic, we can accumulate further quantitative evidence regarding the risk of clustering to consider when designing customized and focused interventions.

## Figures and Tables

**Table 1:** Demographic details of each setting type. The parameters ${\bar{u}}_{x}$, $d_{x}$, $f_{x}$, and $t_{x}$ represent the average number of users per event [persons], the number of days of the event [days], the number of facilities [facilities], and the duration of the event [hours]. The “total number of events”, which indicates how many days the facilities were open/used during the observation period, was calculated by multiplying $d_{x}$ by $f_{x}$. With regard to theaters, the “total number of events”, was calculated as 46,986 [sessions] using statistics rather than calculating from $d_{x}$ and $f_{x}$, for which we could not find reliable data sources.

Category	Establishment Type	Definition of an Event	Type of Users	${\bar{u}}_{x}$ [Users]	$d_{x}$ [Days]	$f_{x}$ [Facilities]	$t_{x}$ [Hours]
Recreational facilities	Theater	Attend a performance	Audiences	341.2	46,986		3.0
	Eating and drinking establishments with Karaoke facility	Visit stores/facilities	Users	110.4	395.0	25,108	2.6
	Sports facility			30.8	268.0	71,271	1.5
	Beauty salon/Barber			6.4	327.0	371,688	1.0
Welfare facility	Welfare facility for persons with disabilities	Stay for a night	Residents, Users	27.5	395.0	5636	16.0
	Elderly care facility			32.2	395.0	28,352	16.0
	Child welfare facility			66.8	268.0	44,616	11.0
Health institute	Hospital	Stay for a night	Inpatients	165.8	395.0	8300	16.0
	Clinic	Visit a doctor	Outpatients	41.1	323.0	102,616	0.5
	Dental clinic			19.7	326.0	68,500	1.0
Eating and drinking establishment	Restaurant	Visit stores	Users	127.4	323.0	1,279,784	1.0
	Alcohol serving eating and drinking establishment			42.9	323.0	264,359	2.0
	Serviced entertaining bars and escort clubs			57.0	323.0	61,857	2.0
Educational institution	University	Attend for a day	Students	3667.4	187.0	795	5.0
	High school			634.4	237.0	4874	8.0
	Middle school			316.6	237.0	10,142	8.0
	Elementary school			322.7	237.0	19,525	7.0
	Kindergarten			111.2	237.0	9698	5.0
	Cram school			122.8	124.7	46,734	1.5
Public office and enterprises	Police station	Work for a day	Staffs	246.2	268.0	1204	8.0
	Local government office			474.1	268.0	1971	8.0
	Fire station			68.2	268.0	2445	8.0
	Workplace			10.6	268.0	5,340,783	7.8

## Data Availability

The monthly cluster incidence by the type of facility is shown in Table S1. Original sources of data and census are included in the reference of the main text and is cited in the supplementary material.

## Supplementary Materials

The following supporting information can be downloaded at: https://www.mdpi.com/article/10.3390/v15020456/s1, Figure S1: Number of reported clusters and cases by month (relative to June 2020); Figure S2: Changes in the number of clusters with the progress of COVID-19 vaccinations; Figure S3: Average number of tests conducted per day by type of tests; Figure S4: Process of annotation of cluster dataset; Figure S5: Flow of data selection; Figure S6: Sensitivity analysis of risk (γ′); Figure S7: Sensitivity analysis of rate ratio in educational institutions; Figure S8: Sensitivity analysis of rate ratio in restaurants; Table S1: Number of COVID-19 clusters by setting types over time (June 2020–June 2021); Table S2: Numerator and Denominators for calculating risks shown in Figure 2; Table S3: Range of parameters for sensitivity analysis; Table S4: Risk Difference of activity-dependent risk of clustering (number of users adjusted, γ′) in restaurants; Table S5: Risk Difference of activity-dependent risk of clustering (number of users adjusted, γ′) in educational institutions; Text S1: Calculation and estimation of parameters. Data sources are presented in the references section of the main text [60,61,62,63,64,65,66,67,68,69,70,71,72,73,74,75,76,77,78,79,80,81,82,83,84,85,86,87,88,89,90,91,92,93].
