# Supplementary material for: Identifying High-Risk Events for COVID-19 Transmission: Estimating the Risk of Clustering Using Nationwide Data
Source: Viruses. 2023 Feb 6;15(2):456. doi: 10.3390/v15020456 (PMC9967753; doi:10.3390/v15020456)
Supplement: Supplementary file 1 [file viruses-15-00456-s001.zip › viruses-2124534-supplementary.pdf]

Supplementary Material for "Identifying high-risk events for COVID-19 transmission: estimating the risk of clustering from nationwide data"

Minami Ueda[a], Katsuma Hayashi[a], Hiroshi Nishiura[a,*]

[a]*Kyoto University School of Public Health, Yoshida-Konoe-cho, Sakyo-ku, Kyoto 606-8501, Japan*

Contents

Figure S1: Number of reported clusters and cases by month (relative to June 2020) **3**

Figure S2: Changes in the number of clusters with the progress of COVID-19 vaccinations **4**

Figure S3: Average number of tests conducted per day by type of tests **5**

Figure S4: Process of annotation of cluster dataset **6**

Figure S5: Flow of data selection **7**

Figure S6: Sensitivity analysis of risk (γ') **8**

Figure S7: Sensitivity analysis of rate ratio in educational institutions **9**

Figure S8: Sensitivity analysis of rate ratio in restaurants **10**

Table S1: Number of COVID-19 clusters by setting types over time (June 2020–June 2021) **10**

Table S2: Numerator and Denominators for calculating risks shown in Figure 2 **12**

Table S3: Range of parameters for sensitivity analysis **12**

*Corresponding author. Kyoto University School of Public Health, Yoshida-Konoe-cho, Sakyo-ku, Kyoto 606-8501, Japan; *Email address:* nishiura.hiroshi.5r@kyoto-u.ac.jp; *Tel.:* +81-75-753-4456.

Table S4: Risk Difference of activity-dependent risk of clustering (number of users adjusted, γ') in restaurants **14**

Table S5: Risk Difference of activity-dependent risk of clustering (number of users adjusted, γ') in educational institutions **14**

Text S1: Calculation and estimation of parameters **14**

---

1. Figure S1: Number of reported clusters and cases by month (relative to June 2020)

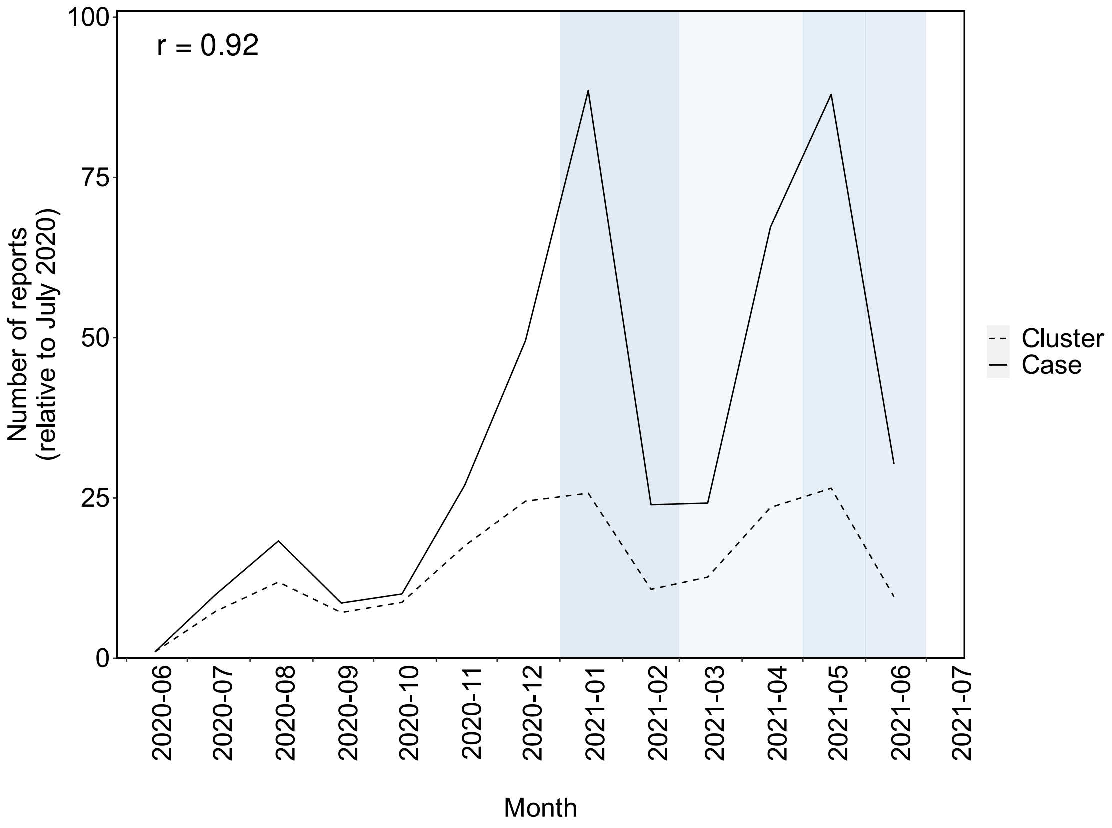

Figure S1: Number of reported clusters and cases by month (relative to June 2020)

The figure compares the number of reported clusters and cases by month, relative to June 2020. Clusters and cases are shown as dashed line and solid line, respectively. Months where State of Emergency (SoE) was declared are shaded in blue. The strength of the shades represent the number of prefectures where SoE was declared: 11 in January and February 2021; 4 in March and April 2021; 10 in May 2021; 9 in June 2021. Correlation coefficient r with respect to the incidence of cluster reports to incidence of confirmed cases is shown in the top left of the figure. Number of cases are based on open data published by the Ministry of Health, Labour and Welfare of Japan.

2. Figure S2: Changes in the number of clusters with the progress of COVID-19 vaccinations

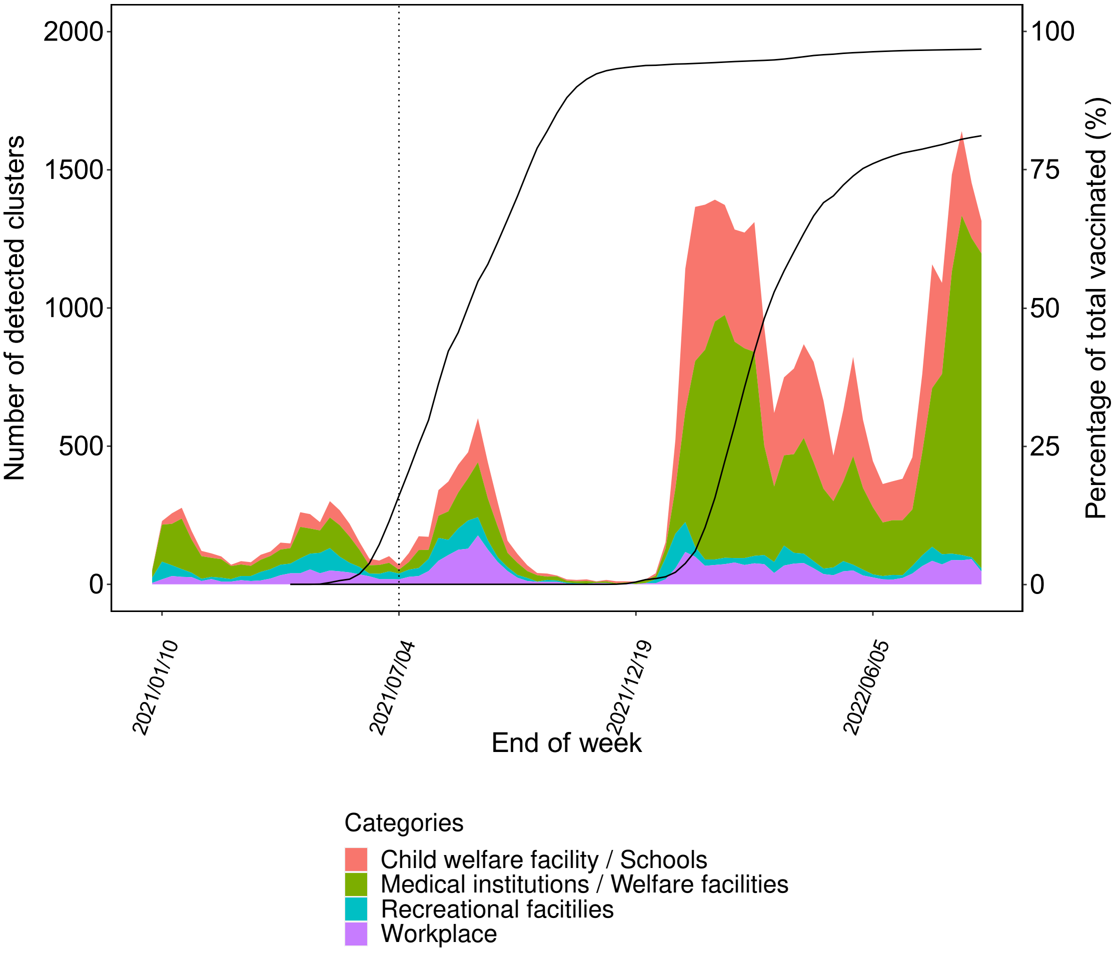

Figure S2: Changes in the number of clusters with the progress of COVID-19 vaccinations

The figure shows the number of clusters from January 2021 to August 2022 (left scale) and the percentage of total vaccinated (right scale). The curves, which represent the percentage of total population, in left and right respectively represents those fully-vaccinated (2 doses of vaccine in total) and boosted (3 doses of vaccine in total). “Child welfare facility / Schools” category includes child welfare facility and all types of educational institutions. “Medical instituions / Welfare facilities” category includes medical institutions, elderly care facilities, and welfare facilities for people with disabilities. “Recreational facilities” includes restaurant, sports facilities, and facilities classified as “others”. “Workplace” includes companies.

3. Figure S3: Average number of tests conducted per day by type of tests

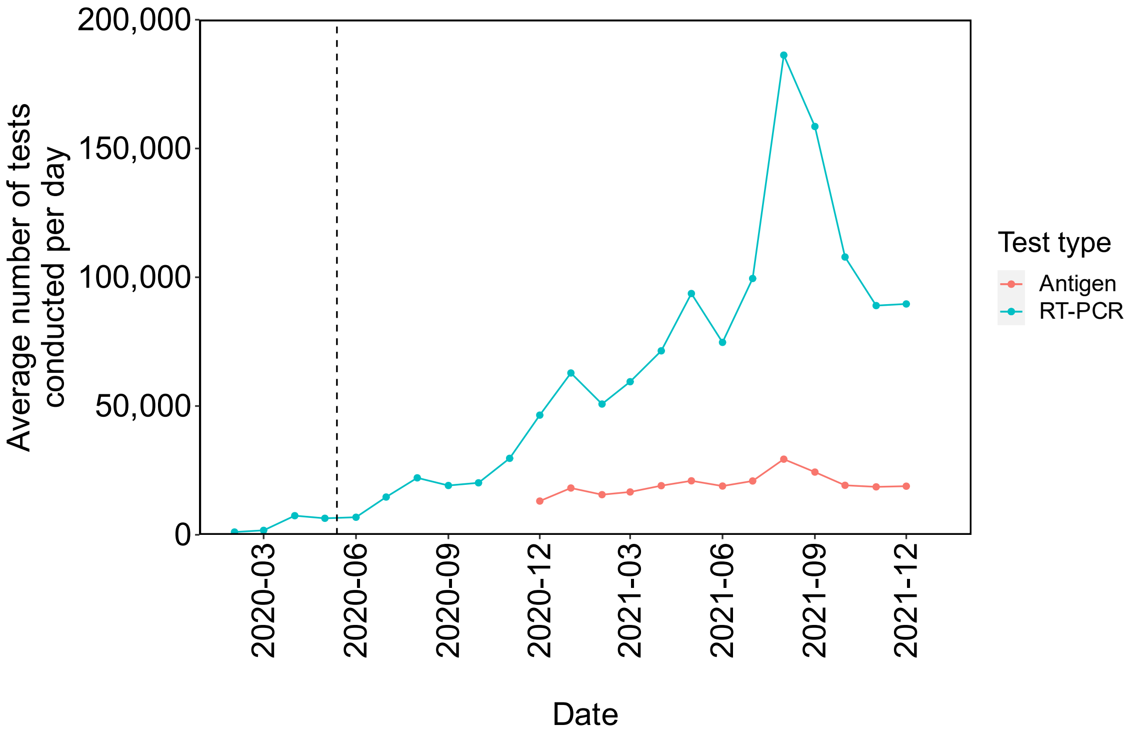

Figure S3: Average number of tests conducted per day by type of tests

The figure represents the average number of tests conducted per day for antigen tests and RT-PCR tests. The number of antigen tests includes both quantitative and qualitative tests. Dashed line represents the day when qualitative antigen test initially recieved pharmaceutical approval in Japan (May 13, 2020). Quantitative antigen test was then approved in June 19, 2020. Number of antigen tests conducted before December 2021 were not available. Datasets released publicly by the Ministry of Health, Labour and Welfare of Japan was used.

4. Figure S4: Process of annotation of cluster dataset

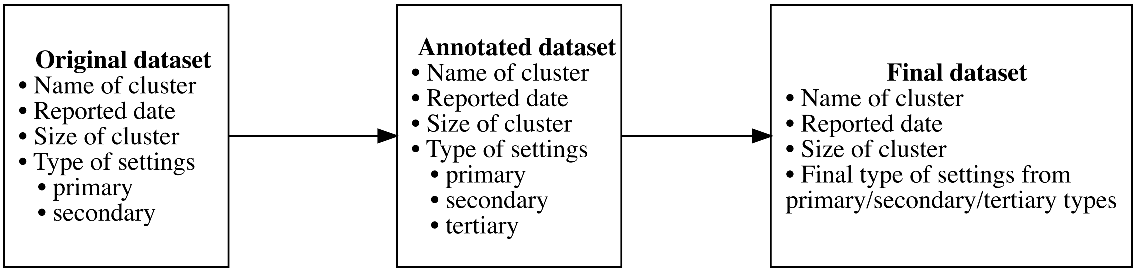

Figure S4: Process of annotation of cluster dataset

The type of setting was already classified into primary and secondary categories in the original data. Since the granularity on the original classification was low, we additionally annotated tertiary and quaternary categories. For example, since schools were only classified as "Schools" in the "Educational Institutions" category, we added type of school such as "High school" as the tertiary category. This enabled the detailed comparison of risks in multiple setting types in the same category.

5. Figure S5: Flow of data selection

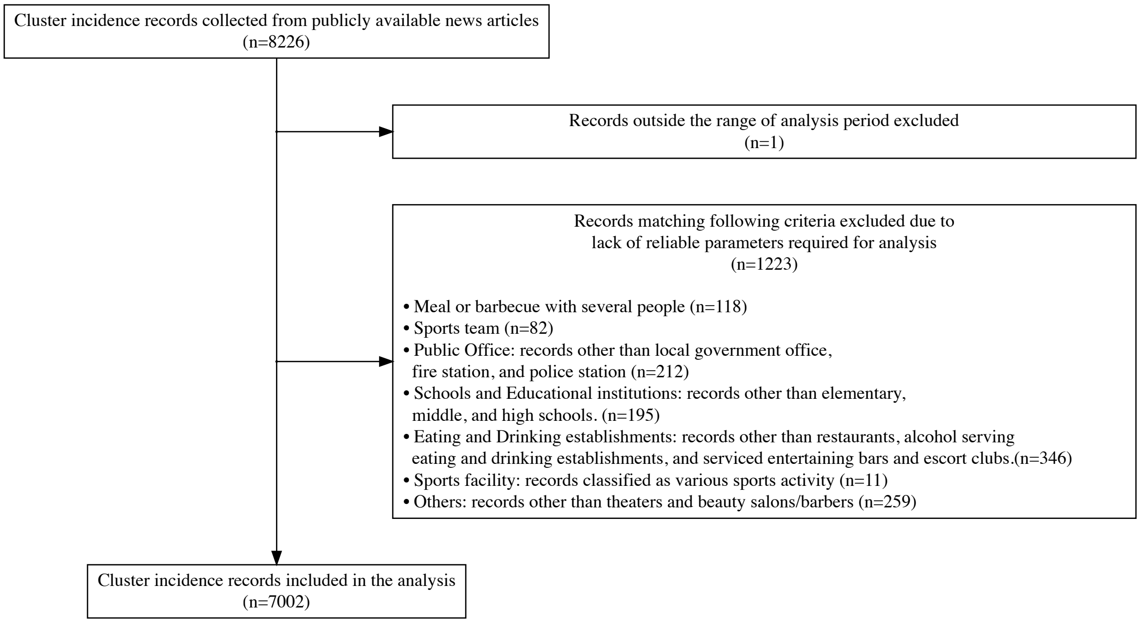

Figure S5: Flow of data selection

After annotating all of the records in the original dataset, we have excluded some records as shown in the figure above. We first excluded a record since it was outside of the study period. Additionally, we excluded 1223 records that matched either of the following criteria: (a) Type of setting with small number of samples; (b) Establishments where "event" could not clearly be defined or calculated (e.g., sports team); (c) Establishments which detailed type of setting could not be identified (e.g., records that are in "School" category but the type of school is unclear).

6. Figure S6: Sensitivity analysis of risk (γ')

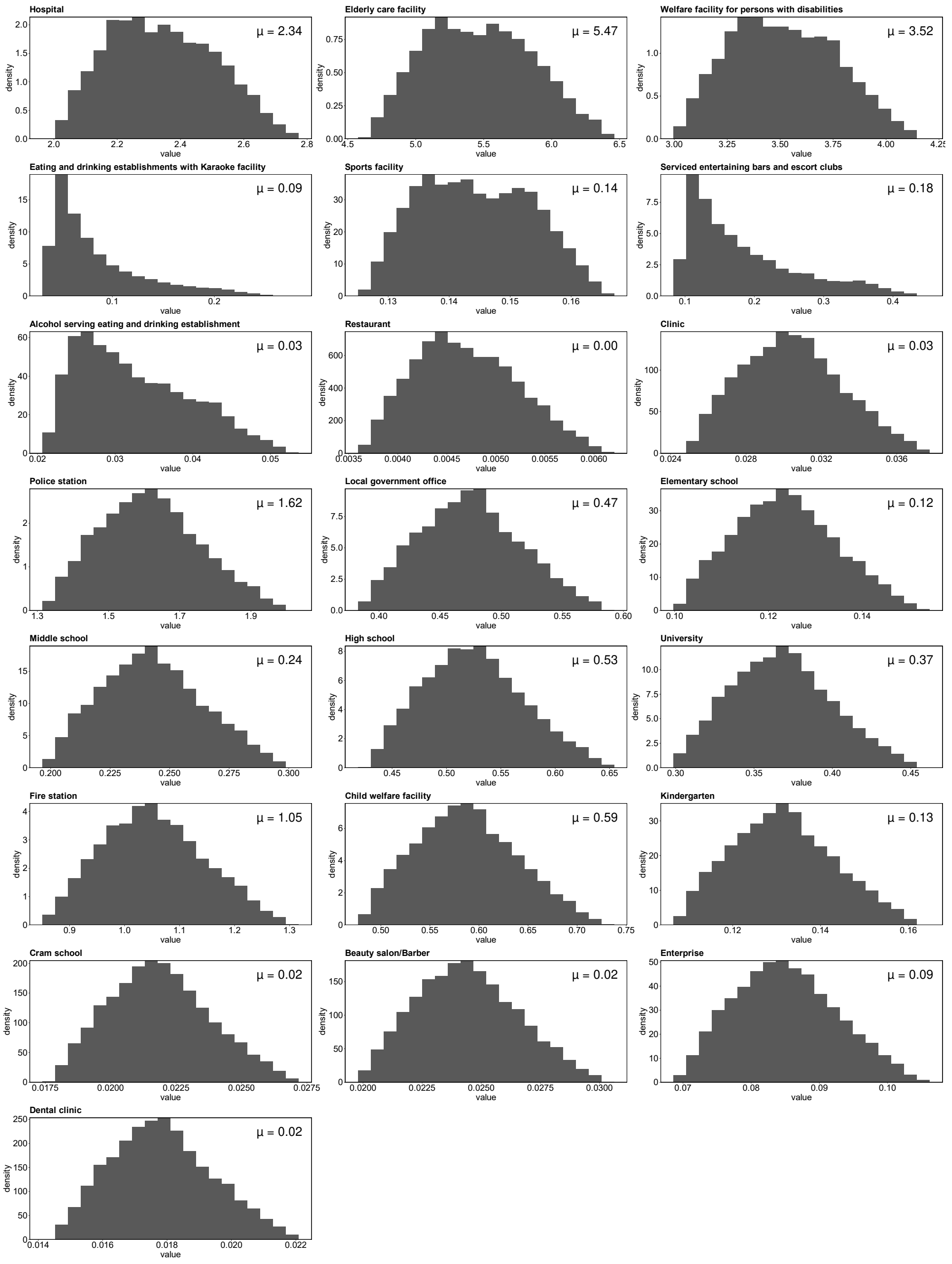

Figure S6: Sensitivity analysis of risk (γ')

7. Figure S7: Sensitivity analysis of rate ratio in educational institutions

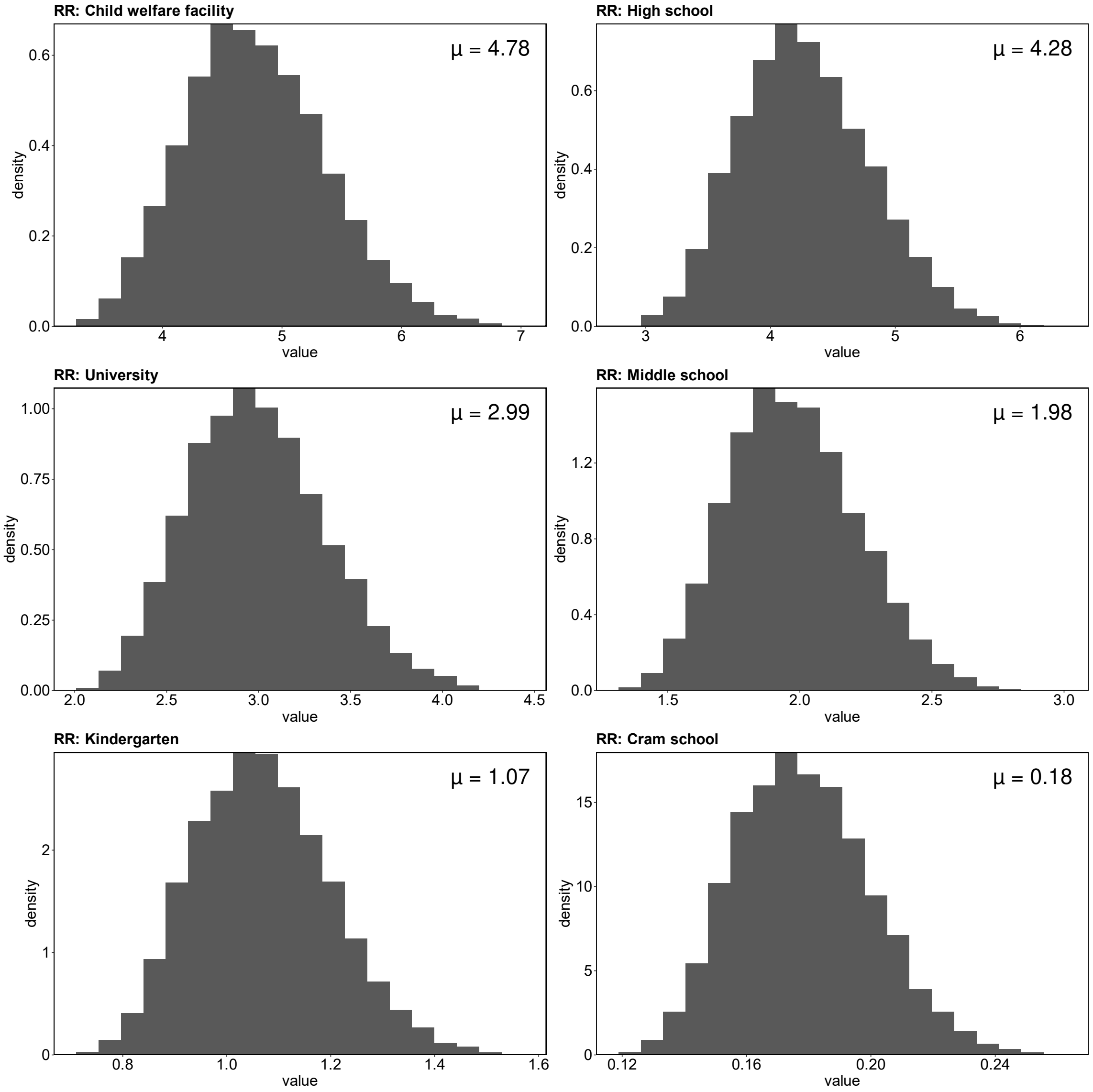

Figure S7: Sensitivity analysis of rate ratio in educational institutions

8. Figure S8: Sensitivity analysis of rate ratio in restaurants

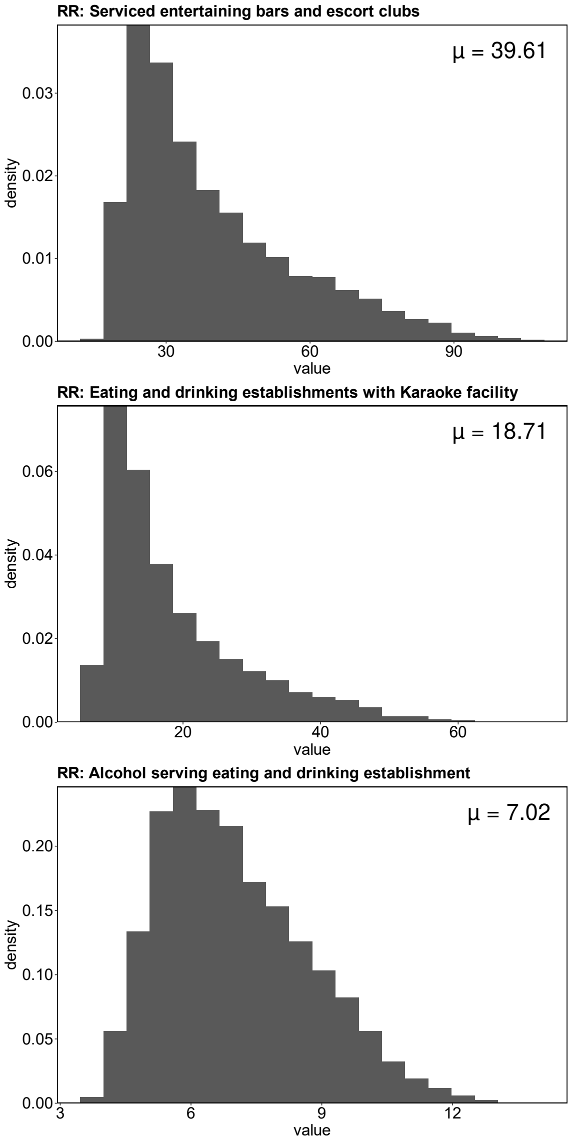

Figure S8: Sensitivity analysis of rate ratio in restaurants

9. Table S1: Number of COVID-19 clusters by setting types over time (June 2020–June 2021)

	2020							2021						
Establishment type	Jun	Jul	Aug	Sep	Oct	Nov	Dec	Jan	Feb	Mar	Apr	May	Jun	**Total** (c_x)
Elderly care facility	3	19	85	32	39	136	220	370	148	95	204	254	69	**1,674**
Enterprise	4	53	57	68	80	95	118	83	55	90	175	178	104	**1,160**
Hospital	7	29	90	37	37	114	169	202	105	82	104	86	23	**1,085**
Serviced entertaining bars and escort clubs	10	41	29	25	40	46	76	47	15	26	44	49	11	**459**
Child welfare facility		12	29	16	12	24	54	45	33	41	50	73	30	**419**
High school		4	19	7	12	28	56	61	9	13	53	63	19	**344**
Restaurant	2	21	29	20	9	22	20	20	6	10	31	56	45	**291**
Karaoke	7	6	24	6	12	21	27	21	8	20	34	37	6	**229**
Welfare facility for people with disabilities		2	8	7	7	21	30	32	9	8	20	27	12	**183**
University	1	11	15	1	7	25	26	10	1	16	35	24	7	**179**
Alcohol serving eating and drinking establishment	1	17	26	6	5	13	22	15	4	19	23	17	6	**174**
Middle school	1	6	6	12	8	21	29	8	6	8	22	24	14	**165**
Elementary school	1	5	3	5	6	34	28	12	14	7	16	25	9	**165**
Police station		3	10	2	8	15	18	21	3	8	12	12	3	**115**
Local government office		5	7	2	4	15	14	12	1	7	15	19	5	**106**
Sports facility		4	4	8	5	6	7	5	2	2	12	23	10	**88**
Fire station		4	1		4	6	6	8	3	2	7	1		**42**
Clinic		1	4			2	7	7	3	1	6	6		**37**
Kindergarten		2	1		1	1	5	3	2	2	2	6	5	**30**
Theater		6	1	2	4	1	2	1				2		**19**
Beauty salon/Barber		3	1	1			3	3		2		2	2	**17**
Cram school			3	1	2		2	1		1	1	2	1	**14**
Dental clinic				1	1			1		1	2	1		**7**
Total	37	254	452	259	303	646	939	988	427	461	868	987	381	**7,002**

Table S1: Number of COVID-19 clusters by setting types over time (June 2020–June 2021)

10. Table S2: Numerator and Denominators for calculating risks shown in Figure 2

Establishment Type x	Numerator (reports)	Denominator of γ_x (event)	Denominator of γ'_x' (person·event)	Denominator of γ''_x (person·event·hour)
Theater	19	46,986	16,033,333	48,100,000
Hospital	1085	3,278,500	543,635,933	8,698,174,920
Elderly care facility	1674	11,199,040	360,264,490	5,764,231,840
Welfare facility for people with disabilities	183	2,226,220	61,158,245	978,531,920
Eating and drinking establishments with Karaoke facility	229	9,917,682	1,095,085,425	2,865,473,528
Sports facility	88	19,100,628	588,195,397	882,293,096
Serviced entertaining bars and escort clubs	459	19,979,811	1,139,428,498	2,278,856,996
Alcohol serving eating and drinking establishment	174	85,387,957	3,663,591,939	7,327,183,878
Restaurant	291	413,370,232	52,668,759,806	52,669,000,000
Clinic	37	33,144,968	1,360,895,900	680,447,950
Police station	115	322,672	79,438,416	635,507,328
Local government office	106	528,228	250,451,628	2,003,613,024
Elementary school	165	4,627,425	1,493,264,241	10,453,000,000
Middle school	165	2,403,654	761,058,903	6,088,471,224
High school	344	1,155,138	732,819,168	5,862,553,344
University	179	148,665	545,218,135	2,736,995,038
Fire station	42	655,260	44,656,304	357,250,432
Child welfare facility	419	11,957,088	798,899,692	8,787,896,612
Kindergarten	30	2,298,426	255,603,552	1,278,017,760
Cram school	14	5,827,808	715,434,095	1,073,151,142
Beauty salon/Barber	17	121,541,976	779,894,346	779,894,346
Workplace	1160	1,431,329,844	15,241,917,368	119,550,000,000
Dental clinic	7	22,331,000	439,350,200	439,350,200

Table S2: Numerator and Denominators for calculating risks shown in Figure 2

11. Table S3: Range of parameters for sensitivity analysis

Establishment type	Number of facilities			Number of users			Number of days			Duration per event		
	base estimate	min	max	base estimate	min	max	base estimate	min	max	base estimate	min	max
Hospital	# registered	fixed	fixed	based on quota	-20%	fixed	open throughout the period	-10%	fixed	assumption*	-20%	fixed
Elderly care facility	# registered	fixed	fixed	based on quota	-20%	fixed	open throughout the period	-10%	fixed	assumption*	-20%	fixed
Welfare facility for people with disabilities	# registered	fixed	fixed	based on quota	-20%	fixed	open throughout the period	-10%	fixed	assumption*	-20%	fixed
Eating and drinking establishments with Karaoke facility	calculated from COVID stat.†	fixed	# from pre-COVID stat.	# from pre-COVID stat.	-20%	fixed	open throughout the period	-10%	fixed	# from pre-COVID stat.	-20%	+20%
Sports facility	# registered	fixed	fixed	# from COVID stat.	fixed	# from pre-COVID stat.	# of days excl. holidays†	-10%	+10%	assumption	-20%	+20%
Serviced entertaining bars and escort clubs	# registered	fixed	fixed	# from COVID stat.	fixed	# from pre-COVID stat.	# of days excl. holidays†	-10%	+10%	assumption	-20%	+20%
Alcohol serving eating and drinking establishment	# registered	fixed	fixed	# from COVID stat.	fixed	# from pre-COVID stat.	# of days excl. holidays†	-10%	+10%	assumption	-20%	+20%
Restaurant	# registered	fixed	fixed	# from COVID stat.	fixed	# from pre-COVID stat.	# of days excl. holidays†	-10%	+10%	assumption	-20%	+20%
Clinic	# registered	fixed	fixed	# from pre-COVID stat.	-20%	fixed	# of days excl. holidays†	-10%	+10%	assumption	-20%	+20%
Police station	# registered	fixed	fixed	based on quota	-20%	fixed	# of days excl. holidays†	-10%	+10%	assumption	-20%	+20%
Local government office	# registered	fixed	fixed	based on quota	-20%	fixed	# of days excl. holidays†	-10%	+10%	assumption	-20%	+20%
Elementary school	# registered	fixed	fixed	based on quota	-20%	fixed	# of days excl. holidays†	-10%	+10%	assumption	-20%	+20%
Middle school	# registered	fixed	fixed	based on quota	-20%	fixed	# of days excl. holidays†	-10%	+10%	assumption	-20%	+20%
High school	# registered	fixed	fixed	based on quota	-20%	fixed	# of days excl. holidays†	-10%	+10%	assumption	-20%	+20%
University	# registered	fixed	fixed	based on quota	-20%	fixed	# of days excl. holidays†	-10%	+10%	# from pre-COVID stat.	-20%	+20%
Fire station	# registered	fixed	fixed	based on quota	-20%	fixed	# of days excl. holidays†	-10%	+10%	assumption	-20%	+20%
Child welfare facility	# registered	fixed	fixed	based on quota	-20%	fixed	# of days excl. holidays†	-10%	+10%	standard nursing hours	-20%	+20%
Kindergarten	# registered	fixed	fixed	based on quota	-20%	fixed	# of days excl. holidays†	-10%	+10%	assumption	-20%	+20%
Cram school	# registered	fixed	fixed	# from pre-COVID stat.	-20%	fixed	# from pre-COVID stat.	-10%	+10%	assumption	-20%	+20%
Beauty salon/Barber	# registered	fixed	fixed	# from pre-COVID stat.	-20%	fixed	# from pre-COVID stat.	-10%	+10%	assumption	-20%	+20%
Enterprise	# registered	fixed	fixed	# from pre-COVID stat.	-20%	fixed	# of days excl. holidays†	-10%	+10%	# from pre-COVID stat.	-20%	+20%
Dental clinic	# registered	fixed	fixed	# from pre-COVID stat.	-20%	fixed	# of days excl. holidays†	-10%	+10%	assumption	-20%	+20%

* All day excluding sleeping hours (8 hours)

† Details are available in Text S1

Table S3: Range of parameters for sensitivity analysis

Each of the parameters were altered within the range presented in the table, assuming a uniform distribution. For base estimates, details of calculations and datasets are presented in Text S1.

12. Table S4: Risk Difference of activity-dependent risk of clustering (number of users adjusted, γ') in restaurants

Establishment Type	Risk Difference	95% CI (lower)	95% CI (upper)
Serviced entertaining bars and escort clubs	0.39731	0.36045	0.43417
Eating and drinking establishments with Karaoke facility	0.20359	0.17650	0.23068
Alcohol serving eating and drinking establishment	0.04197	0.03488	0.04905
Restaurant	0.00000	NA	NA

Table S4: Risk Difference of activity-dependent risk of clustering (number of users adjusted, γ') in restaurants

The table shows the risk difference of activity-dependent risk of clustering (number of users adjusted, γ') in dining occasion setting types, taking "Restaurants" as the baseline.

13. Table S5: Risk Difference of activity-dependent risk of clustering (number of users adjusted, γ') in educational institutions

Establishment Type	Risk Difference	95% CI (lower)	95% CI (upper)
Child welfare facility	0.41398	0.36100	0.46695
High school	0.35892	0.30653	0.41132
University	0.21781	0.16685	0.26878
Middle school	0.10631	0.06918	0.14344
Kindergarten	0.00687	-0.03838	0.05213
Elementary school	0.00000	NA	NA
Cram school	-0.09093	-0.11066	-0.07120

Table S5: Risk Difference of activity-dependent risk of clustering (number of users adjusted, γ') in educational institutions

The table shows the risk difference of activity-dependent risk of clustering (number of users adjusted, γ') in educational institutions, taking "Elementary school" as the baseline.

14. Text S1: Calculation and estimation of parameters

Each of the parameters were calculated using the latest statistics available at the point of analysis.

$\bar{u}_x$: Average number of users per event

- Theater:
  (*Total number of audiences in 2020*[60] [people]) / (*Number of performances held in 2020*[60] [performances])

- Eating and drinking establishment with Karaoke facility:
  (*Average monthly sales per facility*[61] [yen/month]) / (*Average fee per user group*[61] [yen/group]) $*$ (*Average number of users per group*[61] [people/group]) / 30 [days/month]
  The statistics was conducted in 2021.

- Sports facility:
  (*Average number of daily users during data range* [people/day]) / (*Number of facilities* [facilities]). *Average number of daily users* was calculated from the number of monthly users. The number of monthly users at fitness club from June 2020 to June 2021 was available as national statistics (Survey of Selected Service Industries)[62]. For public and private physical exercise facilities, the number of monthly users was estimated chronologically by multiplying the monthly index (which is the comparison of the "Sports facility offering business" of "Indices of Tertiary Industry Activity"[63] to December 2019) respectively to the average number of monthly users calculated from annual statistics from 2017[64,65]. After adding the number of monthly users for all facilities (public physical exercise facility, private physical exercise facility, and fitness club), the average number of daily users was chronologically calculated by dividing by the number of days for each month. The mean was then calculated through the data range as a representative value.

- Beauty Salon/Barber:
  Average number of users per day of beauty salon[66] and barber[67] in 2015 [people/day]

- Elderly care facility:
  (*Number of users at elderly care facilities as of October 2019*[68] [people] + *Number of residents at fee-based homes for the elderly as of October 2019*[68] [people] + *Number of residences with health and welfare services for the elderly as of August 2021*[69] [people]) / (*Number of facilities* [facilities])

- Welfare facility for people with disabilities:
  ADD INFO

- Hospital:

(*Total number of beds as of October 2019*[70] [beds]) ∗ (*Occupancy rate*) / (*Number of facilities* [facilities]). *Occupancy rate* was assumed as 90%.

- Clinic, Dental clinic:
  (*Total number of daily outpatients in all facilities as of 2017*[71] [people]) / (*Number of facilities* [facilities])

- Serviced entertaining bars and escort clubs, alcohol-serving eating and drinking establishment, and restaurants:
  The average number of users per day during the usual period was respectively calculated for each setting type from national statistics as of 2013[72,73]. The number of users for each month was then calculated chronologically by multiplying the ratio of 'Indices of tertiary industry activity'[63] compared to December 2019. Finally, the mean was calculated through the data range as a representative value.

- Police station, fire station, local government office, and enterprise:
  (*Number of staffs*[74–77] [people]) / (*Number of facilities* [facilities])
  The number of staffs for police stations and fire stations are as of 2020. The number of staffs for local government offices is as of 2021. The number of staffs for enterprises is as of 2016.

- Kindergarten, elementary school, middle school, high school, university:
  (*Number of students as of 2020*[78,79] [people]) / (*Number of facilities as of 2020*[78,79] [facilities])

- Child welfare facility:
  (*Number of users as of 2019*[68] [people]) / (*Number of facilities* [facilities])

- Cram school:
  { (*Number of elementary school students as of 2020*[78] [people] ∗ *Rate of cram school attendance*) + (*Number of middle school students as of 2020*[78] [people] ∗ *Rate of cram school attendance*) + (*Number of high school students as of 2020*[78] [people] ∗ *Rate of cram school attendance*) } / (*Number of facilities* [facilities]). *Rate of cram school attendance* for elementary and middle school (46.5% and 61.3%, respectively) was calculated based on the result of a national survey on academics in 2017[80]. *Rate of cram school attendance* for highschool (27.2%) was taken from a statistic report by Benesse Educational Research and Development Institute in 2015[81].

d_x: Number of days

- Sports facility, child welfare facility, police station, fire station, local government office, and enterprise:
  Number of days during data range (June 1, 2020 to June 30, 2021) excluding weekends and national holidays

- Serviced entertaining bars and escort clubs, alcohol-serving eating and drinking establishment, and restaurants:
  Number of days during data range excluding Wednesdays and national holidays

- Beauty salon/Barber:
  Number of days during data range excluding weekends and holidays

- Elderly care facility, welfare facility for people with disabilities, hospital:
  All days during data range

- Clinic:
  Number of days during data range excluding Wednesdays and national holidays

- Dental clinic:
  Number of days during data range excluding Thursdays and national holidays

- Kindergarten, Elementary school, middle school, high school, and university: Number of days during data range excluding weekend, holidays, summer vacation, and spring vacation.
  For kindergarten, elementary, middle, and high school, vacation was regarded as following date range: summer vacation from August 1 to August 20, 2020; winter vacation from December 25, 2020, to January 10, 2021; spring vacation from March 25 to April 5, 2021.
  For university, vacation was regarded as the following: summer vacation from August 1 to September 30, 2021; winter vacation from February 1 to March 31, 2021; no spring vacation.

- Cram school:
  (*Average weekly frequency of cram school attendance* [days/week]) $*$ 4 [weeks/month] $*$ (*Number of months during data range* [month]). *Average weekly frequency of cram school attendance* was obtained by calculating the weighted mean of weekly attendance frequency—taken from a statistics report by Benesse Educational Research and Development Institute in 2015[81]—for elementary, middle, and high school students.

f_x: **Number of facilities**

- Theater: Number of facilities was not used for theater. Instead, the *total number of performances* was alternatively used for the *total number of events*. The total number of performances during data range was estimated by:
  (*Number of performances in 2020*[60] [performances]) / 12 [months] ∗ 13 [months]

- Eating and drinking establishments with Karaoke facility:
  { *Number of Karaoke box stores during usual period (as of 2019)*[61] [facilities] ∗ (1 - (*Temporal closure rate of Karaoke box (as of April 2020)*[82] + *Closure rate of Karaoke box (as of April 2020)*[82])) } + { *Number of bars during usual period (as of 2019)*[61] ∗ (1 - (*Temporal closure rate of bars (as of April 2020)*[82] + *Closure rate of bars (as of April 2020)*[82])) }

- Sports facility:
  (*Number of public physical exercise facilities as of October 2018*[83] [facilities]) + (*Number of private physical exercise facilities as of October 2018*[83] [facilities]) + (*Number of fitness clubs as of October 2020*[84] [facilities])

- Beauty salon/Barber:
  (*Number of beauty salons as of 2019*[85] [facilities]) + (*Number of barbers as of 2019*[85] [facilities])

- Welfare facility for people with disabilities:
  Number of welfare facilities for people with disabilities as of October 2019[68] [facilities]

- Elderly care facility:
  (*Number of elderly care facilities as of October 2019*[68] [facilities]) + (*Number of fee-based homes for the elderly as of October 2019*[68] [facilities]) + (*Number of residences with health and welfare services for the elderly as of August 2021*[69] [facilities])

- Child welfare facility:
  Number of child welfare facilities (Including Certified centers of Early Childhood Education and Care as of October 2019)[68] [facilities]

- Hospital:
  Number of hospitals as of October 2019[70] [facilities]

- Clinic:

  Number of clinics (Dental clinics not included) as of October 2019[70] [facilities]

- Dental clinic:

  Number of dental clinics as of October 2019[70] [facilities]

- Restaurant:

  (*Number of "restaurant" business as of 2019*[85] [facilities] + *Number of "coffee shop business" as of 2019*[85] [facilities]) - (*Number of Serviced entertaining bars and escort clubs* [facilities]) - (*Number of alcohol serving eating and drinking establishment* [facilities])

- Alcohol-serving eating and drinking establishment:

  Number of "late-night liquor service restaurant" as of 2020[86] [facilities]

- Serviced entertaining bars and escort clubs:

  Number of facilities registered as "type 1, 2, 3" under Entertainment Business Act as of 2020[87] [facilities]

- University:

  Number of schools as of 2020[79] [facilities]

- Elementary school, middle school, and high school:

  Number of schools as of 2020[78] [facilities]

- Kindergarten: *Number of kindergartens (Excluding Certified centers of Early Childhood Education and Care) as of 2020*[78] [facilities]

- Cram school:

  Number of cram school business as of 2018[88] [facilities]

- Police station:

  Number of police station as of 2020[89] [facilities] + *Number of police headquarters as of 2020*[89] [facilities]

- Local government office:

  Number of local government office (prefectures and municipalities) as of October 2021[90] [facilities]

- Fire station:
  (*Number of fire station as of 2020*[75] [facilities]) + (*Number of fire defense headquarters as of 2020*[75] [facilities])

- Enterprise:
  Number of enterprise office as of 2016[77] [facilities]

t_x**: Duration per event**

- Theater:
  Estimated under the assumption of a typical duration of a theater performance

- Eating and drinking establishment with Karaoke facility:
  Statistics from White Paper on Karaoke[61]

- Sports facility, Beauty Salon/Barber, Clinic, Dental clinic, Serviced entertaining bars and escort clubs, alcohol-serving eating and drinking establishment, restaurants, and cram school:
  Estimated under the assumption of typical use case

- Elderly care facility, Welfare facility for people with disabilities, and Hospital:
  Assumed as whole day excluding 8 hours of sleep

- Police station, fire station, local government office:
  Assumed work time as 9:00–17:00

- Enterprise:
  Statistics from Ministry of Health, Labor, and Welfare[91]

- Kindergarten:
  Assumed timetable as 9:00–14:00

- Elementary school:
  Assumed timetable as 8:30–15:30

- Middle school and high school:
  Assumed timetable as 8:30–16:30

- University: Statistics from Benesse Educational Research and Development Institute:
  Total hours spent at the university per week (hours)[92] / 5 (days)

- Child welfare facility:

  Standard nursing hours, defined by ministerial ordinance article four[93]
